# Qualifying choice: ethical reflection on the scope of prenatal screening

**DOI:** 10.1007/s11019-016-9725-2

**Published:** 2016-09-08

**Authors:** Greg Stapleton

**Affiliations:** Department of Health, Ethics and Society, Faculty of Health, Medicine and Life Sciences, GROW School for Oncology and Developmental Biology, Maastricht University, 6200 MD Maastricht, The Netherlands

**Keywords:** Abortion, Ethics, Non-invasive prenatal testing, Prenatal screening, Reproductive autonomy, Reproductive choice

## Abstract

In the near future developments in non-invasive prenatal testing (NIPT) may soon provide couples with the opportunity to test for and diagnose a much broader range of heritable and congenital conditions than has previously been possible. Inevitably, this has prompted much ethical debate on the possible implications of NIPT for providing couples with opportunities for reproductive choice by way of routine prenatal screening. In view of the possibility to test for a significantly broader range of genetic conditions with NIPT, the European Society of Human Genetics (ESHG) and American Society of Human Genetics (ASHG) recommend that, pending further debate, prenatal screening for reproductive choice should only be offered where concerning serious congenital conditions and childhood disorders. In support of this recommendation, the ESHG and ASHG discuss a number of ethical issues on which they prompt further debate: the informational privacy of the future child, the trivialization of abortion, the risk of information overload, and issues of distributive justice. This paper responds to this call with further reflection on each ethical issue and how it relates to the moral justification of providing couples with opportunities for *meaningful* reproductive choice. The paper concludes that whilst there may be good reasons for qualifying the scope of any unsolicited prenatal screening offer to serious congenital conditions and childhood disorders, if prenatal screening is justified for providing couples with opportunities for *meaningful* reproductive choice, then health services may have obligations to empower couples with the same opportunity where concerning other conditions.

## Introduction

Many health services provide some type of prenatal screening service that is routinely offered to women and their partners during antenatal care. Although there is much plurality in policy and practice between countries, it is common that a minimum set of screening options are provided. Typically, these include screening options for infectious diseases such as HIV, Syphilis, and Hepatitis B. They also include screening options for clinical conditions, namely rhesus D incompatibility, gestational diabetes, and pre-eclampsia. In these cases, screening is offered for the purpose of improving clinical outcomes for both the mother and the future child through the early detection of disease and timely provision of preventative treatment or therapy (HCN 2008; NICE 2008). Accordingly, participation in screening is often presented as a matter of course by the clinician. Yet, many health services offer an additional set of screening options where this would be inappropriate. These latter screening options address heritable and congenital conditions for which preventative treatment or therapy has limited effect or is unavailable altogether. This usually includes the following conditions: the trisomies Down syndrome (T21), Edward syndrome (T18), and Patau syndrome (T13); the hemoglobinopathies sickle cell disease and thalassemia; as well as structural anomalies including neural tube defects such as anencephaly and spina bifida (Godard et al. 2003a, b; HCN 2008; NICE 2008). When screening is offered for these conditions international guidelines recommend that it should instead be aimed at providing couples with opportunities for reproductive choice of whether or not to have a child with a serious medical disorder (NCB 1993, 2006; HCN 2008).

Up until recently, the range of heritable and congenital conditions for which prenatal screening could be offered remained somewhat limited. However, this is set to change significantly with the prospect that genome wide fetal profiling may soon be possible with Non-invasive Prenatal Testing (NIPT) (Wong and Dennis Lo 2016). Couples may then use NIPT to test for, diagnose, and make reproductive choices about a much broader range of (genetic) conditions. Not only might this be achievable much earlier during pregnancy than is presently possible but it may furthermore allow couples to circumvent the risk of iatrogenic miscarriage associated with using invasive diagnostic techniques amniocentesis or chorionic villi sampling, upon which current prenatal screening pathways are reliant (Alfirevic et al. 2003; de Jong et al. 2011; Tabor and Alfirevic 2010; Wong and Dennis Lo 2016).

In view of these developments, the European Society of Human Genetics (ESHG) and American Society of Human Genetics (ASHG) recommend that, pending further debate, prenatal screening should only be offered for reproductive choice where concerning serious congenital and heritable disorders that affect childhood (Dondorp et al. 2015). In support of this tentative recommendation, the ESHG and ASHG raise a number of ethical issues on which they prompt further debate: the informational privacy of the future child, the trivialization of abortion, the risk of information overload, and issues of distributive justice. This paper aims to respond to this call with further reflection on each ethical issue and how it relates to the moral justification of providing couples with opportunities for meaningful reproductive choice. The paper is primarily concerned with whether or not the structural directivity of the proposed scope for prenatal screening is justified in view of the aim of providing couples with opportunities for *meaningful* reproductive choice; conceptualized within this paper as informed and autonomous reproductive choices of whether or not to continue pregnancy, that enable couples to avoid suffering they anticipate for themselves and/or their future child (NCB 1993, 2006; de Jong and de Wert 2015; HCN 2008). The following research questions are explored: what is the principle moral justification for providing couples with the opportunity for reproductive choice in the format of routine prenatal screening, where concerning fetal anomalies such as Down syndrome and neural tube defects? How might the justification apply to other conditions for which NIPT could soon be available? Is this consistent with the tentative recommendations presented within the joint ESHG and ASHG position paper on NIPT?

## Prenatal screening for fetal anomalies

For most forms of screening, participants benefit from the early detection of disease and timely provision of preventative treatment or therapy. However, this may not always be the case where concerning screening for fetal anomalies such as Down syndrome and neural tube defects. For these conditions preventative treatment or therapy may have only a limited effect or is perhaps unavailable altogether. As a consequence, couples may receive few practical courses of action other than to decide whether or not to terminate the pregnancy. This has prompted considerable debate over why prenatal screening for conditions that are generally not preventable might be offered in the first place (Clarke 1997; de Jong and de Wert 2015; Juth and Munthe 2012; Munthe 2015; van El et al. 2012; Wilkinson 2015). In relation to this question, several justifications are readily discussed within ethical debate. Generally speaking, these correspond to one of two conflicting frameworks: (1) where preferred reproductive choices may be promoted by the health service provider (a directive framework) and (2) where preferred reproductive choices should not be promoted by the health service provider (a non-directive framework). In the following section, justifications associated with either framework will be discussed.

### The directive framework

During the early development of prenatal screening programmes that targeted fetal anomalies, prenatal screening was routinely offered for objectives that implied that couples *should* participate in order to avoid the birth of an affected child (Centerwall 1970; Navon and Padeh 1971; Stein 1975; Stein and Susser 1971). This directive approach has been justified for three principle reasons. First, selectively aborting fetuses with conditions for which prenatal and perinatal prevention is generally not possible could help to avoid suffering for the future child (Clarke 1997; Green 1997). The emphasis of this appeal is on preventing future persons from having to endure particularly severe physical and psychological suffering that might be associated with some heritable or congenital disorders. Clarkeburn (2000) argues that parents who are aware they are at risk may have moral (non-legal) obligations to participate in prenatal screening where concerning severe health conditions for which many might consider life not worth living. Clarkeburn suggests that only for conditions characterized by significant levels of intellectual disability and continuous non-palliative pain would it be reasonable to believe that non-existence is in the best interests of the future child (2000).

The appeal to ‘avoid suffering’ is also used in another justification for offering prenatal screening. However, within this second justification, the concept of suffering does not relate to the wellbeing of the future child, but instead, concerns the psychosocial health of prospective parents and their family. The main concern relates to the anguish and grief that a couple may experience as parents of a child whose suffering cannot be prevented (Clarke 1997). However, unlike the previous appeal to avoid the suffering of the future child within this latter justification women have no moral obligations to consider screening. The offer may instead be viewed as a form of paternalism. This justification is often cited in support of offering screening for conditions that might qualify as a life not worth living. However, it may theoretically be applied to any condition where it is reasonable to expect significant levels of distress may be experienced by prospective parents. For example, the principle is also relevant in cases where couples (and their families) may primarily feel burdened by obligations towards providing care and support for their child, rather than, by the child’s suffering (Faden et al. 1987; Lippman 1991).

The third justification for offering screening for reproductive choice is that this may lessen the overall burden of disease on society (Clarke 1997; Juth and Munthe 2012; Stein 1975; Stein and Susser 1971; Wilkinson 2015). Unlike the personal appeals to avoid suffering, justifications based on the social utility of women’s reproductive choices are highly impersonal and only indirectly concerned with the wellbeing of each couple and their future child. The primary concern is that screening is organized in a way that maximizes its benefit to society (Wilkinson 2015). An extreme application of this principle can be seen within some economic evaluations of prenatal screening programmes. For example, in a critical review of the economic appraisal literature, Mooney and Lange (1993) raise concern about the use of models that derive benefit from women electing to terminate an affected pregnancy (i.e. it is assumed there is no benefit from screening if women do not abort affected fetuses). Such models differ in their assessment of the women’s utility based on the condition for which screening is offered and the number of ‘healthy’ replacement pregnancies occurring after an abortion (e.g. 0, 1, or 1 < X). Mooney and Lange point out that within these models (1993): “*[benefits] take the form of various savings in public expenditure (e.g. health services), in family expenditure on the child, in avoided lost maternal output and the child’s consumption of other goods and services*” (1993, p. 874). Accordingly, greater levels of directivity may be preferred when offering screening for conditions which require more expensive medical care and social support.

### Counter perspectives

Objections to offering prenatal screening for fetal anomalies generally focus on the disproportionality of benefits and harms received by different stakeholders when preferred reproductive choices are promoted by the health service provider. These objections are associated with fetal rights, feminist, and disability rights perspectives (de Jong and de Wert 2015; Johnsen 1986; Parens and Asch 2003; Wertz and Fletcher 1993). From the fetal rights perspectives, the life of the unborn fetus is considered sacred. Abortion is therefore inherently wrong. Since prenatal screening ‘for reproductive choice’ provides couples with an ‘opportunity’ for an abortion, health services are thought to be complicit in this wrong. Whilst this applies to the provision of any opportunities for reproductive choice, when the opportunity is presented in a way that promotes the use of abortion (e.g. such as within an unsolicited screening offer as opposed to following personal enquiry) health services hold a higher level of responsibility for wrong doing. Screening for reproductive choice is therefore less objectionable when it does not influence the autonomy of couples’ reproductive decisions. From the most extreme fetal rights perspective, the fetus has a moral status equivalent to that of any adult person. Accordingly, abortion is generally viewed as the moral equivalent of murder. Screening for reproductive choice would therefore be highly problematic in all but a few very rare cases where either the life of the expectant mother is threatened or the life of the fetus would not be considered worth living (Clarkeburn 2000). Although this position is sometimes dismissed as religious dogma, it has been argued by appealing to the similarity of both developmental origins and potential futures jointly shared by the fetus and adult person (Gill 2005; Marquis 1989). In contrast, more moderate perspectives assign a lower moral status to the fetus based on growing biological and psychological similarities between the fetus, newborn, and adult. From this less radical perspective, the moral status of the fetus gradually increases throughout its development. Yet, greater emphasis is placed on later stages of pregnancy (Gillespie 1977; Steinbock 2011). Whilst abortion is still considered to be a controversy option, a considerably more lenient view is taken on when it might be acceptable, and therefore, when prenatal screening for reproductive choice might also be acceptable.

The disability rights perspective is primarily concerned with the issue of bias (Kaposy 2013; Reinders 2000). More specifically, why screening for reproductive choice might be offered for some conditions but not for others? In the context of prenatal screening for Down syndrome, some families affected by Down syndrome have argued that the condition does not prevent them from leading worthwhile and fulfilling lives. It has been suggested that the most significant source of suffering for many affected families is stigma, discrimination, and the general lack of inclusiveness within society (Brasington 2007; Cunningham 1996). In cases where suffering may be avoided through social interventions, offering screening for reproductive choice would not appear to be about serving the needs of the future child and/or prospective parents, but rather, about lessening the burden of disease on ‘the rest’ of society. If screening for reproductive choice is offered for this reason, it conveys a discriminatory message about people living with the condition (Holm 2008; Parens and Asch 2003). For example, in their article on the preventability of Down syndrome, Stein and Susser suggest that prenatal diagnostic testing should be offered to older pregnant women as one of four preventative measures to reduce the incidence of Down syndrome among new born populations. In support of this position, they problematize the increasing longevity of people living with Down syndrome, stating that “*But whatever is done, the survivors continue in a state of permanent dependence that imposes a severe burden on their families and on existing forms of social organization.”* (Stein and Susser 1971, p. 650). They clarify “*The goal of public health in such a situation must be prevention, and preferably primary prevention, that is, the reduction of the incidence of the disorder by action taken before it becomes manifest.*” (p. 651). Whilst it may be unintended, these statements convey a discriminatory message: ‘people’ with Down syndrome are a burden on ‘the rest’ of society, and therefore, unwelcome. However, prejudice may not always be conveyed overtly. Offering screening for reproductive choice may still be problematic if health services are organized inequitably. This might apply to screening that is offered in the context of diminishing investment in care and support for affected families, or alternatively, if access to abortion services is conditionally linked to a diagnosis of disability.

From a feminist perspective, any offer of prenatal screening for reproductive choice where preferred reproductive choices are recommended by the health service provider may threaten women’s reproductive rights and freedoms (Johnsen 1986). Reproductive decision making, especially when concerning abortion, should remain a voluntary and highly personal practice that reflects the biological role that women play during reproduction and takes into account the way that women experience pregnancy (Dondorp et al. 2015; Lippman 1991; HCN 2008; Rothman 1986; Wertz and Fletcher 1993). Any suggestion that women should participate in screening in order to avoid the birth of an affected child might pressure women into distressing and emotionally burdensome decisions which they may later come to regret. Such pressure might not only be the result of an explicit recommendation. It is also possible that health policy contributes to a coercive social context for making reproductive choices. For example, providing access to abortion services only in cases of disability or reducing investment in care and support for affected families may lead to social pressures towards making certain reproductive choices and not others. However, the feminist position is not categorically opposed to the offer of screening for reproductive choice. Instead it is suggested that many women positively value the opportunity that an offer of prenatal screening provides (Lippman 1991; van Schendel et al. 2014; Wertz and Fletcher 1993).

### The non-directive framework

In view of these objections, international guidelines now recommend that screening for reproductive choice should only be offered within a framework of non-directivity. If screening for reproductive choice is offered within a non-directive framework it would seem much harder to claim that health services convey a discriminatory message about individuals with disabilities or promote the subjugation of women’s reproductive autonomy. This position is endorsed within guidelines for responsible screening published by health authorities in the UK, the Netherlands, and within many other Western countries (de Jong et al. 2011; Godard et al. 2003a, b; HCN 2008). The two most characteristic features of this framework are that health services should adopt a position of neutrality with respect to the outcomes of couples’ reproductive choices (i.e. there are no preferred pre- or post-test choices) and should support couples in making informed and autonomous reproductive choices in line with their own values of whether or not to have an affected child (HCN 2008). For example, in the report ‘*Screening: Between hope and hype*’, commissioned by the Health council of the Netherlands, it is argued that although screening may not always benefit participants in terms of improved health outcomes, participants may still derive some (personal) utility through the provision of reliable information upon which an informed and autonomous reproductive choice can be made (2008). This concept of utility differs from that adopted within the directive framework which is mainly focused on the utility of reproductive outcomes. The directive framework is broadly based on normative criteria first articulated by Wilson and Junger in 1968, before prenatal testing for fetal anomalies was widely available (Andermann et al. 2008; Wilson and Jungner 1968). Wilson and Junger endorse the use of a conservative concept of utility that is primarily about preventing disease through early detection and timely provision of treatment or therapy. Screening programmes with this aim are typically assessed in terms of the overall reduction in mortality, morbidity, and quality of life. In contrast, the concept of utility applied within the non-directive framework is about providing couples with opportunities for *meaningful* reproductive choice; generally understood as informed and autonomous reproductive choices of whether or not to continue with pregnancy, that enable couples to avoid suffering they anticipate for themselves and/or their future child (de Jong et al. 2011; Dondorp et al. 2010; Wilfond and Thomson 2000).

## Ethical challenges

As argued by de Jong and de Wert, moral objections to offering prenatal screening are weaker when it is the couple and not the state that decides whether or not to avoid the birth of an affected child (2015). To ensure that health services remain impartial with respect to couples’ (pre- and post-test) reproductive choices whilst also supporting them in making *meaningful* reproductive choices, international guidelines recommend that several criteria should be met. First, health services should make it clear to couples that prenatal screening is offered in order to provide them with an opportunity for making meaningful reproductive choices about whether or not to continue with an affected pregnancy and that they may freely decide to accept or decline the offer. This recommendation applies most critically to communications between the physician and the expectant couple. However, it is also intended to apply to policy, educational materials, and other documentation in which the purpose of screening may be addressed. The main ethical concerns are that women and their partners are fully informed of the aim of screening, that they may be confronted with challenging information, and that they understand that they are free to make their own reproductive choices. Second, additional services that actively support couples in making *meaningful* reproductive choices should also be provided. In this respect, health services should ensure the quality of screening options offered, provide non-directive pre- and post-test counseling, provide educational support, and maintain equitable access to follow-up services (e.g. services for abortion and services that provide care and support to affected families) (NCB 1993, 2006; HCN 2008; PCEPBR 1983).

Although there is broad consensus that where prenatal screening for reproductive choice is offered it should be offered within a non-directive framework, at present there remains much debate as to whether principles of non-directivity should also be applied to the scope of prenatal screening. With respect to this issue, ethical debate on the offer of prenatal screening appears polarized between providing couples with ‘pure choice’ and ‘qualified choice’ in what to screen for (de Jong and de Wert 2015; Munthe 2015; Wilkinson 2015). For proponents of pure choice, non-directivity is applied in its most absolute sense. The structural directivity of an unsolicited, yet, qualified prenatal screening offer ‘for the purpose of reproductive choice’ is therefore problematic (for reasons conveyed by fetal rights, feminist, and disability rights perspectives). In contrast, for proponents of qualified choice, the structural directivity of a qualified offer is justifiable as long as couples are still able to make meaningful reproductive choices about whether or not to have an affected child. As a result, some variant of the non-directive framework is used to determine which screening options should be offered. In relation to this issue, the ESHG and ASHG discuss several ethical issues in support of offering prenatal screening only where concerning serious congenital and childhood disorders: the informational privacy of the future child, the trivialization of abortion, the risk of information overload, and issues of distributive justice (Dondorp et al. 2015). These ethical issues will now be discussed within the following commentary where justifications for qualified choice will be examined with respect to the principle justification for offering prenatal screening; to provide couples with opportunities for meaningful reproductive choice.

### The informational privacy of the future child

The idea of limiting the scope of screening in order to protect the informational privacy of the future child relates to the concern that children may (later on) learn of personal health information that was attained by their parents during prenatal screening. The main ethical issue is that children may experience psychosocial distress from foreknowledge that they may later develop a disorder for which (primary) prevention is unavailable. The breach of informational privacy is sometimes discussed in terms of a violation of the child’s right to an open future (i.e. to choose for themselves whether to know about the condition) (Andorno 2004; BSHG 2010; Wright 2009). Although this concern is primarily associated with screening for adult onset conditions, it is also relevant for sub-clinical conditions that might go undiagnosed during childhood but the label of which may still cause some psychosocial harm or impinge upon a person’s right to informational privacy (Dondorp et al. 2015).

Whilst the idea of protecting the future child from psychosocial and informational harm has strong moral appeal, it is not fully apparent how it should be taken into account in view of the aim of offering prenatal screening. For example, why might any appeal to protect the informational privacy of a (possible) future child represent a more convincing objection to couples’ having the opportunity to make meaningful reproductive choices about adult onset conditions and sub-clinical conditions, than the appeal to protect the presumed interests of the (existing) fetus in becoming that future child? If providing couples with the opportunity for meaningful reproductive choice through routine prenatal screening is justifiable for any condition where the life of the (possible) future child does not qualify as a life not worth living, health services must presumably adopt one of the following assumptions: either the fetus has no ‘interests’ to be considered, which will include any future privacy interests, or alternatively, the interests of the fetus are conditional on couples’ meaningful reproductive choices, in which case, health services will have a *pro tanto* reason to discount them when offering prenatal screening. Certainly there is a *conditional* risk associated with participating in prenatal screening for adult onset conditions and sub-clinical conditions; a risk that is entirely contingent on couples electing not to terminate an affected pregnancy following prenatal screening. This represents a challenging ethical issue. Since any risk to the future child will be conditional on couples’ reproductive choices, not offering prenatal screening to protect (possible) future children may imply that health services are not aiming to provide couples with opportunities for reproductive choices that are meaningful to them for avoiding suffering.

In view of the conditionality of the risk to the future child, some directivity with respect to the safe use of prenatal screening for adult onset conditions and sub-clinical conditions would seem justifiable in order to discourage couples from participating, for example, if they do not intend to avoid the birth of an affected child (Bunnik et al. 2013). For these conditions, the aim of prenatal screening should be more about ensuring that couples have the opportunity to make a meaningful pre-test reproductive choice that takes into account the seriousness of any conditional risk to the future child. It would therefore seem appropriate that these screening options are not offered within the same normative framework used to offer other prenatal screening options that do not carry a conditional risk to the future child. When considering this issue, it could be justifiable to refrain from making an unsolicited offer of screening for adult onset and sub-clinical conditions during routine antenatal care. Yet, if health services *genuinely* aim to provide couples with the opportunity to make meaningful reproductive choices, then health services may have obligations to ensure that couples are similarly empowered where concerning adult onset and sub-clinical conditions. One way in which this might be achieved would be to only offer such opportunities following personal enquiry. In this respect, the offer would no longer be unsolicited yet couples that anticipate suffering for themselves and/or their future child may still be provided with an opportunity for making a meaningful reproductive choice about adult onset and sub-clinical conditions. If such an approach would be adopted, it would seem advisable to ensure that couples’ have sufficient capabilities to anticipate suffering for themselves and/or their future child where concerning adult onset and sub-clinical conditions. A practical solution to this issue would be to develop strategies that engage couples prior to conception in order to more effectively educate and counsel them on their opportunities in preparation for pregnancy.

### Information overload

Although there are different interpretations of what information should be provided in order to attain informed consent, there is general agreement that health services should present couples with relevant information about (1) the characteristics of any condition for which screening is offered, (2) the characteristics of any screening tests that are offered, and (3) the implications of any test results that may follow (van den Berg et al. 2005). Accordingly, as the scope of screening expands the amount of information that each couple must process is likely to increase. The central ethical issue here is the risk of uninformed reproductive choice. Couples who become confused, distressed, or overwhelmed by the level or complexity of post-test information provided may be unable to make choices that are consistent with their own values. However, information overload is also an important pre-test concern during the offer of screening, where there is an additional risk that couples may simply become burdened by the level of choice on offer (Bunnik et al. 2013). Couples that experience this issue may be unable to fully understand the implications of their pre-test choices and the risks associated with participating in screening.

Given that the risk of information overload is likely to be exacerbated if more numerous kinds of conditions with dissimilar implications are included within the scope of screening, there is a strong moral imperative to prioritize conditions to be included within the scope of prenatal screening. Yet, at the same time, limiting the scope of screening in order to reduce the risk of informational overload may also increase its structural directivity. This places significant moral importance on what criterion might be used to include or exclude a condition from the scope of screening and why. In relation to this issue, it would not be appropriate to ‘rank’ conditions according to the utility that avoiding the birth of an affected child may have for couples (Mooney and Lange 1993). Although this may be intuitive, assuming a priori which reproductive outcomes would avert most suffering conflicts with the principle aim of providing couples with opportunities for meaningful reproductive choice. In line with principles of non-directivity, health services should not convey the message that terminating an affected pregnancy will benefit either the prospective parents or future child in terms of avoiding suffering. Although this would be considered least problematic where concerning conditions that qualify as a life not worth living, the issue remains that couples should have discretion to make such value judgements for themselves. The principle concern here is that the ‘seriousness’ of each reproductive outcome for avoiding suffering should be determined by each couple in view of their own personal situation and not assumed by the health service on behalf of all couples.

Yet, it would seem unproblematic to assess screening options according to couples’ informational needs where concerning the implications of each reproductive outcome for the future child and for themselves (Pergament and Pergament 2012). For example, screening for conditions that are characterized by more severe levels of intellectual disability and greater levels of continuous non-palliative pain may provide couples with a strong indicator of the quality of life of the future child, and thus, be more informative. Screening for conditions where there are more significant obligations for parents, in terms of the provision of care and support, may also be more useful. Whereas, screening for conditions where quality of life of the future child is more significantly determined by individually affecting social factors may be least useful to couples. Since most couples will be familiar with such factors before the offer of prenatal screening they may already have sufficient capabilities to make meaningful reproductive choices about them. Therefore, an unsolicited screening offer may provide them with little added value over and above making prenatal screening services for the same conditions available upon request.

### The trivialization of abortion

In a study of public viewpoints, Farrimond and Kelly report that *“…fears about trivialisation are linked to the rejection of* ‘*picking and choosing’ and a valuation of natural diversity such as disability. As such, trivialisation fears are not fears about having greater information per se, but are rather the fear of the ‘trivialisation of abortion’ (de Jong* et al*. 2010)”* (2013, p. 740). Trivialization fears appear to represent a general concern that screening for reproductive choice may empower couples in using abortion for unimportant (i.e. not for avoiding suffering) or for discriminatory reasons. Concerns have also been raised over the additional harms that ‘trivial’ reproductive choices may have in society, such as a loss of ‘natural’ diversity or a perceived public endorsement of discriminatory views. Such harms are primarily referenced in objections to offering screening for non-medical traits (Hall et al. 2009; Wright 2009). Although it would be preferable to avoid these harms, it is difficult to understand why they might only be problematic when screening is offered for non-medical traits. For example, evidence indicates that some women who engage in screening for the purpose of sex-selection may do so in order to avoid suffering they anticipate for themselves and/or their future child (Puri et al. 2011; Raphael 2002; Wertz and Fletcher 1998). It may therefore not be so apparent that a couple’s reproductive choices where concerning non-medical traits are always unimportant. Yet, if the main ethical concern is that the offer of screening may falsely convey an endorsement of discriminatory views, then it would be necessary to explain why this might be unproblematic where concerning conditions for which screening might still be offered (e.g. Down syndrome).

A possible defense to this latter charge might be to argue that the public only perceive such an ‘endorsement’ when screening is offered for conditions that conflict with their own norms and values relating to ‘important’ reproductive choices. Within a recent attitudinal study on NIPT, Dutch women suggested that they preferred the idea of offering screening for *“severe or fatal disorders that could lead to the early death of a child or to a very low quality of life”* (van Schendel et al. 2014, p. 1349). Van Schendel et al. further report that “*Participants also feared a so*-*called ‘slippery slope’, which could lead to people starting to test for minor abnormalities, gender or for cosmetic traits like blond hair and blue eyes*.*”* (van Schendel et al. 2014, p. 1348). If such views are indeed widely shared then it could be argued that only where screening is offered for non-medical traits would a discriminatory message be conveyed. Yet, noting that discrimination which may only be perceived by minority groups is discounted within such an argument, additional inconsistencies are also apparent.

Although findings from attitudinal studies indicate that screening for serious medical conditions is widely preferred, it is not clear that all participants are quite so opposed to couples receiving greater levels of individual choice. Van Schendel et al. states that *“[the participants] argued that even though it might be possible to determine whether an unborn child has a severe disorder, a prenatal test like NIPT cannot predict its severity or the quality of life of the child. Moreover, participant’s stated that quality of life is a relatively subjective concept and differs per person, which all makes it very difficult to decide whether to test and to continue with the pregnancy or not. Nevertheless, many participants felt that women should be able to make their own decision about what to test for and what not to test for”* (van Schendel et al. 2014, p. 1349). A more radical acceptance of individual choice is evidenced in a study by Farrimond and Kelly who note that for a minority of their participants “*There is a clear prioritisation of parental choice about NIPD (67:* +*5): ‘it should be the parents’ decision what tests to have and what they want to do with the results’ (P22, female, currently pregnant).”* (Farrimond and Kelly 2013, p. 739). Farrimond and Kelly go on to clarify that: “*Furthermore, they agree with expanding testing to include sex determination and testing for non*-*medical conditions, both rejected in all other factors (4:* +6*; 7: −*5): “it’s their choice to make” (P10, female, two children)*.” (Farrimond and Kelly 2013, p. 739). In view of these findings, it is less obvious that social norms and values will conflict quite so strongly with the full range of screening options that could be offered.

Perhaps a more reasonable objection to offering screening for non-medical traits may be possible which neither discounts any discrimination that may be perceived by minority groups nor implies that a couple’s reproductive choices are always trivial where concerning such conditions. In view of reported findings from attitudinal research in Western countries, it would seem that the majority of couples would not wish to use prenatal screening where concerning non-medical traits for the purpose of avoiding the birth of an affected child (Faden et al. 1987; Harrington et al. 1996; van Schendel et al. 2014). A much more reasonable complaint may then be that routinely offering screening for the purpose of reproductive choice where concerning non-medical traits, may burden important antenatal services that should be prioritized for couples with greater need of them. The more prominent role of personal, individually affecting, social determinants of suffering for non-medical traits, means that couples are unlikely to benefit from an unsolicited screening offer (and the provision of associated services for educational support and counseling) over and above making prenatal screening available following personal enquiry. In this respect, a more reasonable objection to routinely offering screening for non-medical traits might be that this could ‘trivialize’ the provision of an important antenatal service and undermine public solidarity towards providing couples with opportunities for meaningful reproductive choice.

### Issues of distributive justice

The main issues of distributive justice discussed within the bioethics literature relate to the question of whether or not the use of scarce public resources for the provision of prenatal screening is justifiable in view of public health priorities. Debate of these issues is primarily polarized by an ethical tension between conflicting moral imperatives that both serve the aim of providing couples with opportunities for meaningful reproductive choice. Within the bioethics literature there is broad consensus that a publicly funded prenatal screening programme aimed at promoting ‘pure choice’ is unjustifiable (Clarke 1997; de Jong and de Wert 2015; Munthe 2015; Wilkinson 2015). This is contested for two principle reasons. The first relates to ethical issues that have been discussed previously within this paper: Offering pure choice could enable couples to make trivial reproductive choices (the trivialization of abortion), reproductive choices with that may harm future children (the informational privacy of the future child), or result in uninformed reproductive choices (information overload). In view of these ethical issues, de Jong and de Wert argue that prenatal screening should instead be aimed at “*enabling individual pregnant women (and their partners) to make meaningful reproductive choices with regard to having or not having a child with a serious disorder or disability.*” (2015, p. 50). De Jong and de Wert clarify that “*This can be seen as a combination of the second and third candidate goals of prenatal screening as distinguished by Clarke [1. Spare public resources; 2. Avoidance of suffering; 3. Promotion of informed reproductive choices], qualifying the latter (no ‘pure autonomy’) and adding to the former that ‘avoidance of suffering’ need not only refer to possible suffering of the future child, but may as well refer to the impact of the birth of a child with a disorder or handicap on the life of the woman, the couple, or the family.”* (2015, p. 50). Although this is currently the preferred normative framework in many Western countries (HCN 2008), concerns have been raised that qualifying choice to ‘serious disorders or disabilities’ will not adequately reflect the heterogeneity of meaningful reproductive choices that couples may wish to make given the opportunity (Dondorp et al. 2015; de Jong and de Wert 2015; Munthe 2015). This point is further emphasized when considering some of the problems associated with arguments supporting qualified choice that have been discussed previously within this paper. Screening may therefore incorporate a structural directivity that conflicts with the objective of providing couples with informed and autonomous reproductive choices that are meaningful *to them* for avoiding suffering. This may be especially problematic within contexts where screening for conditions that fall beyond the scope of publicly funded prenatal screening programmes is also unavailable privately.

The second issue relates to the problem of offering pure choice responsibly within a context of resource constraint. The costs associated with offering pure choice within a morally justifiable framework are thought to be prohibitive and generally unjustifiable in view of opportunity costs (e.g. funding the provision of care and support for people affected by serious disorders or disabilities). Munthe suggests that offering pure choice may only be ethically responsible when health services ensure equal levels of access and basic knowledge about services, require greater initiative to be taken by couples in seeking and requesting prenatal screening, and maintain adequate levels of non-directive pre- and post-test counseling (Munthe 2015). Munthe points out that “*It will be very expensive to maintain the required adaptability of testing*-*kits and sufficient standards of counselling. Mere promotion of reproductive autonomy will hardly serve to justify such costs in a public priority*-*setting context*. *Focusing on the new PNT [Prenatal Testing] as a source of liberation and self*-*determination thus rather drives a notion of it as a reproductive information technology to be used by people outside publicly funded services.”* (2015, p. 43). However, it has been noted that inequalities in opportunity for meaningful reproductive choice are likely to develop if access to prenatal screening is left to commercial providers (de Jong and de Wert 2015; Munthe 2015; HCN 2008). Such inequalities would seem especially problematic where concerning more serious disorders and disabilities, over which the majority of couples appear to be concerned (Faden et al. 1987; Harrington et al. 1996; van Schendel et al. 2014). When considering the strong preference towards more serious medical conditions, the number of couples that will need to access prenatal screening for meaningful reproductive choice outside of a public funded screening programme targeted at serious disorders and disabilities is likely to be minimal.

Ethical debate on issues of distributive justice reveals conflicting moral imperatives that both serve the goal of providing couples with opportunities for meaningful reproductive choice. On the one hand, prioritizing more serious medical disorders may serve the majority’s needs yet at the same time it may increase the structural directivity of any publicly funded prenatal screening service. On the other hand, broadening the scope may lessen the structural directivity of screening but may burden services that facilitate couples in making meaningful reproductive choices and expend scarce public resources that could be used to tackle public health priorities (such as providing care and support for families affected by serious disorders or disabilities). Presently, there appears to be no ‘ideal’ criteria readily available that may be used to balance these competing imperatives. Whilst prenatal screening is organized under the ethos of providing couples with opportunities for meaningful reproductive choice, the structural directivity associated with qualifying choice to include only serious congenital and childhood disorders is likely to remain a controversial practice. However, such ethical tensions may be resolved if the offer of prenatal screening is instead aimed at empowering couples with sufficient capabilities for making meaningful reproductive choices. Implied by this aim is that the scope of screening *should not be* about promoting meaningful reproductive choices as understood by the health service provider or general public, but rather, *should be* about ensuring that couples are sufficiently capable of anticipating (and avoiding) suffering for themselves and/or their future child. Another (significant) implication of this framework is that couples should have the opportunity to make meaningful reproductive choices about conditions that fall beyond the recommended scope of serious congenital and childhood conditions. Further ethical debate is necessary on how access to such opportunities may be provided and whether there may be *pro tanto* reasons for prohibiting it within certain social contexts.

## Concluding remarks

Whilst collectively, ethical challenges associated with the informational privacy of the future child, information overload, the trivialization of abortion, and issues of distributive justice may provide good reasons for qualifying the scope of any routine prenatal screening offer to serious congenital conditions and childhood disorders, they do not represent coherent moral objections to providing couples with the opportunity for making meaningful reproductive choices about other conditions. If the use of public health resources is justified for providing couples with opportunities for meaningful reproductive choice through the offer of prenatal screening, then public health services may have obligations to similarly empower couples where concerning conditions that fall beyond the scope of serious congenital conditions and childhood disorders. Ensuring that couples have opportunities for making meaningful reproductive choices about conditions for which prenatal screening is not routinely offered may lessen ethical tensions relating to the structural directivity of the offer. One way in which this could be achieved would be to offer couples the opportunity to screen for such conditions following some form of personal enquiry. Couples that anticipate suffering for themselves and/or their future child may then have the opportunity for making meaningful reproductive choices. If such an approach were to be adopted, it would seem advisable to develop strategies to educate and counsel couples prior to conception in order to ensure that couples have sufficient capabilities to anticipate suffering for themselves and/or their future child. Inevitably resource constraints, and limits set by the risk of information overload, will require priority setting. Whilst prenatal screening is offered for the purpose of providing couples with opportunities for meaningful reproductive choice, qualifying choice to serious congenital and childhood disorders is likely to remain a controversial practice. Ethical tensions associated with the structural directivity of qualified choice may however be resolved if prenatal screening is instead aimed at empowering couples with sufficient capabilities for making meaningful reproductive choices. This alternative position appears more compatible with an ethos of non-directivity yet also justifies the structural directivity of qualified choice.
